
JOURNAL INFORMATION
==============================
NLM Title Abbreviation: ScientificWorldJournal
Journal ID: ScientificWorldJournal
NLM Journal ID: 101131163
Journal ID: TSWJ

The Scientific World Journal

ISSN: 2356-6140
EISSN: 1537-744X
Publisher: Wiley

ARTICLE INFORMATION
==============================
PMCID: PMC5956399
PMID: 15349515
DOI: 10.1100/tsw.2004.127
Article ID: 576408
Article version: 1
Subjects: Case Study

Pseudotumoral Aspect of the Hydatic Cyst of the Kidney

Sarf Ismail *
Meziane Anas
El Mejjad Amine
Taha Abdellatif
Aboutaeib Rachid
Meziane Fathi
CHU IBN Rochd, Department of Urology, Casablanca, Morocco
*Ismail Sarf: sarf.ismail@caramail.comAcademic Editor: Anthony Atala

Publication date: 2004
Electronic publication date: 2004 Aug 30
Volume: 4
First page: 758
Last page: 759
Received 2004 Apr 28; Revised 2004 Aug 12; Accepted 2004 Aug 30
Copyright: Copyright © 2004 Ismail Sarf et al.
Copyright year: 2004
License: This is an open access article distributed under the Creative Commons Attribution License, which permits unrestricted use, distribution, and reproduction in any medium, provided the original work is properly cited.
License URL: https://creativecommons.org/licenses/by/3.0/

Keywords: hydatid cyst, kidney, C.T scan, nephrectomy

==============================
Case Study
*TheScientificWorldJOURNAL* (2004) 4, 758–759
ISSN 1537-744X; DOI 10.1100/tsw.2004.127

### Pseudotumoral Aspect of the Hydatic Cyst of the Kidney

Ismail Sarf*, Anas Meziane, Amine El Mejjad, Abdellatif Taha, Rachid Aboutaeib, and Fathi Meziane
*CHU IBN Rochd, Department of Urology, Casablanca, Morocco*

**E-mail:** sarf.ismail@caramail.com

*Received April 28, 2004; Accepted August 12, 2004; Published August 30, 2004*

**KEYWORDS:** hydatid cyst, kidney, C.T scan, nephrectomy

**DOMAIN:** urology

### CASE REPORT

A 58-year-old woman was examined for isolated continuous pains in the right flank since 3 months. She had no specific urinary problems. The physical exam she underwent did not detect any fullness in the right flank.

Serum biochemistry, haematology, and urine analysis were all normal. The ultrasound exam showed a heterogeneous mass of the right kidney. The computed tomography (CT) revealed a right renal mass measuring 4 cm out of 5 cm, having a central necrosis, few calcifications on the wall, and a heterogeneous enhancement by contrast product (Fig. 1). The diagnosis of the right renal carcinoma was highly suspected, then a right nephrectomy was performed.

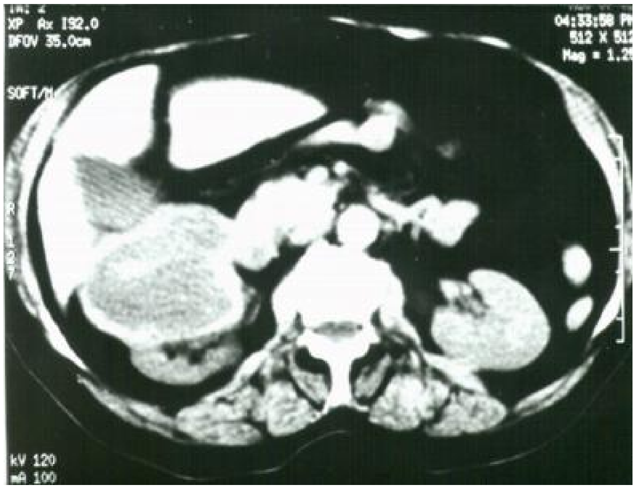

**FIGURE 1.** Computed tomography: Right renal mass 4/5 cm, with central necrosis and heterogeneous enhancement by contrast product.

*Corresponding author. Tel: 00 212 64043110
©2004 with author.

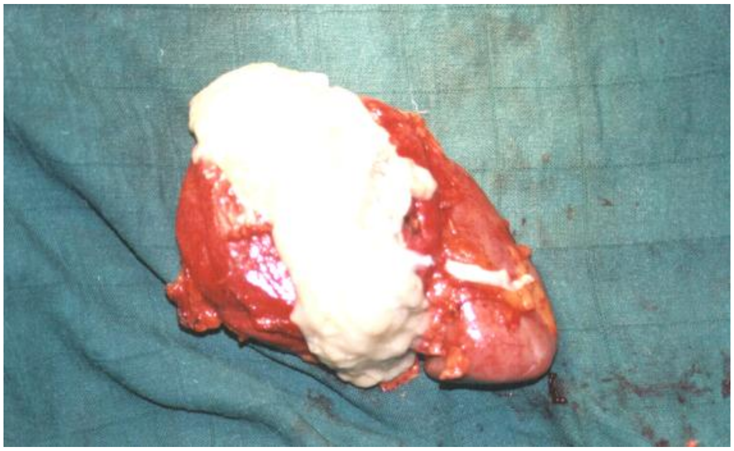

**FIGURE 2.** Macroscopic aspect: presence of whitish gelatinous infected substance at the opening of the kidney.

The macroscopic aspect of the kidney at its opening showed a whitish gelatinous infected substance with no evidence of malignant formation (Fig. 2).

The pathological examination showed a quasi-damaged kidney containing an atypical cyst full of hydatic membranes with thick wall.

### COMMENT

Hydatic disease of the kidney is an unusual entity, representing 2–4% of all cases of hydatic disease[1]. An accurate preoperative diagnosis of a hydatic cyst of the kidney assumes a high level of suspicion for the entity, since it usually mimics more familiar lesions of the kidney[2].

CT is assumed to be the most diagnostic of all laboratory and radiological investigations[3]. However, through this case, the authors think that the CT scan may be misleading and want to draw to the attention of all clinicians serving in the endemic area that hydatic disease should be considered in the differential diagnosis of solid occupying urinary tract lesions.

### REFERENCES

1. Kirkland, K. (1966) Urological aspects of hydatic disease. *Br. J. Urol*. **39,** 241.
2. Diamond, H.M., Lyon, E.S., and Hui, N.T. (1976) Echinococcal disease of the kidney. *J. Urol.* **115,** 742.
3. Afsar, H., Yagci, F., and Meto, S. (1994) Hydatic disease of the kidney: evaluation and features of diagnostic procedures. *J. Urol*. **151,** 567.

**This article should be referenced as follows:**

Sarf, I., Meziane, A., El Mejjad, A., Taha, A., Aboutaeib, R., and Meziane, F. (2004) Pseudotumoral aspect of the hydatic cyst of the kidney. *TheScientificWorldJOURNAL* **4**, 758–759.

**Handling Editor:**

Anthony Atala, Principal Editor for *Urology* — a domain of *TheScientificWorldJOURNAL*.
